# Ultra-processed food consumption and semen quality parameters in the Led-Fertyl study

**DOI:** 10.1093/hropen/hoae001

**Published:** 2024-01-17

**Authors:** Cristina Valle-Hita, Albert Salas-Huetos, María Fernández de la Puente, María Ángeles Martínez, Silvia Canudas, Antoni Palau-Galindo, Cristina Mestres, José María Manzanares, Michelle M Murphy, Montse Marquès, Jordi Salas-Salvadó, Nancy Babio

**Affiliations:** Universitat Rovira i Virgili, Departament de Bioquímica i Biotecnologia, Alimentació, Nutrició, Desenvolupament i Salut Mental (ANUT-DSM), Reus, Spain; Institut d’Investigació Sanitària Pere Virgili (IISPV), Reus, Spain; Centro de Investigación Biomédica en Red de Fisiopatología de la Obesidad y Nutrición, Instituto de Salud Carlos III, Madrid, Spain; Institut d’Investigació Sanitària Pere Virgili (IISPV), Reus, Spain; Centro de Investigación Biomédica en Red de Fisiopatología de la Obesidad y Nutrición, Instituto de Salud Carlos III, Madrid, Spain; Universitat Rovira i Virgili, Departament de Ciències Mèdiques Bàsiques, Unitat de Medicina Preventiva, Alimentació, Nutrició, Desenvolupament i Salut Mental ANUT-DSM, Reus, Spain; Department of Nutrition, Harvard T.H. Chan School of Public Health, Harvard University, Boston, MA, USA; Universitat Rovira i Virgili, Departament de Bioquímica i Biotecnologia, Alimentació, Nutrició, Desenvolupament i Salut Mental (ANUT-DSM), Reus, Spain; Institut d’Investigació Sanitària Pere Virgili (IISPV), Reus, Spain; Centro de Investigación Biomédica en Red de Fisiopatología de la Obesidad y Nutrición, Instituto de Salud Carlos III, Madrid, Spain; Universitat Rovira i Virgili, Departament de Bioquímica i Biotecnologia, Alimentació, Nutrició, Desenvolupament i Salut Mental (ANUT-DSM), Reus, Spain; Institut d’Investigació Sanitària Pere Virgili (IISPV), Reus, Spain; Centro de Investigación Biomédica en Red de Fisiopatología de la Obesidad y Nutrición, Instituto de Salud Carlos III, Madrid, Spain; Department of Nutrition, Food Sciences and Gastronomy, School of Pharmacy and Food Sciences, Food Torribera Campus, University of Barcelona, Santa Coloma de Gramenet, Spain; Institute of Nutrition and Food Safety of the University of Barcelona, INSA-UB Maria de Maeztu Unit of Excellence, Santa Coloma de Gramenet, Spain; Universitat Rovira i Virgili, Departament de Bioquímica i Biotecnologia, Alimentació, Nutrició, Desenvolupament i Salut Mental (ANUT-DSM), Reus, Spain; Institut d’Investigació Sanitària Pere Virgili (IISPV), Reus, Spain; Centro de Investigación Biomédica en Red de Fisiopatología de la Obesidad y Nutrición, Instituto de Salud Carlos III, Madrid, Spain; ABS Reus V. Centre d’Assistència Primària Marià Fortuny, Salut Sant Joan de Reus—Baix Camp, Reus, Spain; Universitat Rovira i Virgili, Departament de Bioquímica i Biotecnologia, Alimentació, Nutrició, Desenvolupament i Salut Mental (ANUT-DSM), Reus, Spain; Institut d’Investigació Sanitària Pere Virgili (IISPV), Reus, Spain; ABS Reus V. Centre d’Assistència Primària Marià Fortuny, Salut Sant Joan de Reus—Baix Camp, Reus, Spain; Institut d’Investigació Sanitària Pere Virgili (IISPV), Reus, Spain; Hospital Universitari Sant Joan de Reus, Universitat Rovira i Virgili, IISPV, Alimentació, Nutrició, Desenvolupament i Salut Mental ANUT-DSM, Reus, Spain; Institut d’Investigació Sanitària Pere Virgili (IISPV), Reus, Spain; Centro de Investigación Biomédica en Red de Fisiopatología de la Obesidad y Nutrición, Instituto de Salud Carlos III, Madrid, Spain; Universitat Rovira i Virgili, Departament de Ciències Mèdiques Bàsiques, Unitat de Medicina Preventiva, Alimentació, Nutrició, Desenvolupament i Salut Mental ANUT-DSM, Reus, Spain; Institut d’Investigació Sanitària Pere Virgili (IISPV), Reus, Spain; Center of Environmental, Food and Toxicological Technology—TecnATox, Rovira i Virgili University, Reus, Spain; Universitat Rovira i Virgili, Departament de Bioquímica i Biotecnologia, Alimentació, Nutrició, Desenvolupament i Salut Mental (ANUT-DSM), Reus, Spain; Institut d’Investigació Sanitària Pere Virgili (IISPV), Reus, Spain; Centro de Investigación Biomédica en Red de Fisiopatología de la Obesidad y Nutrición, Instituto de Salud Carlos III, Madrid, Spain; Universitat Rovira i Virgili, Departament de Bioquímica i Biotecnologia, Alimentació, Nutrició, Desenvolupament i Salut Mental (ANUT-DSM), Reus, Spain; Institut d’Investigació Sanitària Pere Virgili (IISPV), Reus, Spain; Centro de Investigación Biomédica en Red de Fisiopatología de la Obesidad y Nutrición, Instituto de Salud Carlos III, Madrid, Spain

**Keywords:** ultra-processed food, semen quality, diet, male infertility, cross-sectional, lifestyle, reproductive age, Led-Fertyl study

## Abstract

**STUDY QUESTION:**

Is ultra-processed food (UPF) consumption associated with semen quality parameters?

**SUMMARY ANSWER:**

Higher UPF consumption was inversely associated with total sperm count, sperm concentration, and total motility in men of reproductive age.

**WHAT IS KNOWN ALREADY:**

The consumption of UPF, which has been rising during the last decades, has been demonstrated to be positively associated with several chronic diseases such as diabetes or cardiovascular diseases. However, the scientific evidence on its potential impact on semen quality remains notably limited.

**STUDY DESIGN, SIZE, DURATION:**

A cross-sectional analysis was conducted using data from 200 healthy men (mean age 28.4 ± 5.5 years) enrolled in the Led-Fertyl (Lifestyle and Environmental Determinants of Seminogram and Other Male Fertility-Related Parameters) study between February 2021 and April 2023.

**PARTICIPANTS/MATERIALS, SETTING, METHODS:**

UPF consumption (% of energy from UPF) was estimated according to the NOVA classification system using a validated 143-item semi-quantitative food frequency questionnaire. Total sperm count, sperm concentration, sperm vitality, total motility, progressive motility, and normal sperm forms were set as the main outcomes. Microscopic parameters were analyzed using a phase-contrast microscope and a computer-assisted sperm analysis (CASA) system. Semen samples were collected and tested according to World Health Organization 2010 standards. Multivariable linear regression models were fitted to estimate the associations between UPF tertile and semen quality parameters.

**MAIN RESULTS AND THE ROLE OF CHANCE:**

Sperm concentration (*β*: −1.42 × 10^6^ spz./ml; 95% CI: −2.72 to −0.12) and motility (*β*: −7.83%; 95% CI: −15.16 to −0.51) were lower in participants in the highest tertile of UPF compared to the lowest. A similar association was observed for sperm count when UPF was analyzed per 10% increment of energy from UPF consumption (*β*: −1.50 × 10^6^ spz.; 95% CI: −2.83 to −0.17). Theoretically replacing 10% of energy from UPF consumption with 10% of energy from unprocessed or minimally processed food consumption was associated with a higher total sperm count, sperm concentration, total motility, progressive motility, and normal sperm forms.

**LIMITATIONS, REASONS FOR CAUTION:**

Cross-sectional studies do not permit the drawing of causal inferences. Measurement errors and reporting bias cannot be entirely ruled out.

**WIDER IMPLICATIONS OF THE FINDINGS:**

This work suggests that consumption of UPF may have an impact on certain semen quality parameters. Furthermore, opting for unprocessed or minimally processed foods instead of UPFs could potentially benefit semen quality. If these results are replicated in future epidemiological studies with different long-term designs, these novel findings could provide valuable insights for updating or even designing preventive and interventional programs to address infertility among men of reproductive age.

**STUDY FUNDING/COMPETING INTEREST(S):**

This study was supported by the Spanish government’s official funding agency for biomedical research, ISCIII, through the Fondo de Investigación para la Salud (FIS), the European Union ERDF/ESF, ‘A way to make Europe’/‘Investing in your future’ [PI21/01447], and the Diputació de Tarragona (2021/11-No.Exp. 8004330008-2021-0022642). J.S.-S. gratefully acknowledges the financial support of ICREA under the ICREA Academia program. C.V.-H. received a predoctoral grant from the Generalitat de Catalunya (2022 FI_B100108). M.Á.M. was supported by the Sara Borrell postdoctoral fellowship (CD21/00045—Instituto de Salud Carlos III (ISCIII)). M.F.d.l.P. was supported by a predoctoral grant from the Rovira i Virgili University and Diputació de Tarragona (2020-PMF-PIPF-8). All authors have no conflict of interest to declare.

**TRIAL REGISTRATION NUMBER:**

N/A.

WHAT DOES THIS MEAN FOR PATIENTS? Infertility affects approximately 8–12% of couples of reproductive age, with male factors contributing to up to 40–50% of this burden. Environmental and lifestyle factors appear to play a significant role in semen quality. Specifically, adhering to a healthy diet rich in unprocessed or minimally processed foods, such as fruits, vegetables, legumes, or nuts, while limiting the consumption of red and processed meat and sugar-sweetened beverages, has been associated with improved semen quality.

Unfortunately, there has been a rapid increase in the consumption of ultra-processed foods (UPFs) in recent years. These foods are characterized by their poor nutritional quality and the presence of various added ingredients such as sugar, salt, fat, and additives. They have also been linked to several chronic diseases, including diabetes, hypertension, cardiovascular disease, and cancer. However, it remains unclear whether the consumption of UPFs is related to poorer semen quality parameters.

To address this gap in the field of nutrition and semen quality, we conducted a study involving 200 healthy men of reproductive age. Our findings suggest that a higher consumption of UPFs is associated with poorer semen quality, as indicated by reduced total sperm count, sperm concentration, and total motility. In addition, substituting the consumption of UPFs with unprocessed or minimally processed foods was mostly associated with improved semen quality parameters. Nevertheless, further research with long follow-up periods is necessary to confirm our observations and explore the underlying biological mechanisms behind these associations.

## Introduction

There is growing concern regarding infertility and human semen quality because 8–12% of couples of reproductive age, around the world, have difficulties conceiving. It is estimated that male factors account for up to 40–50% of this infertility burden (Agarwal *et al.*, 2021). The remarkable decrease in semen quality over the last decades, particularly in developed and industrialized countries, highlights the potential roles of environmental and lifestyle factors in this decline (Levine *et al.*, 2017; Vander Borght and Wyns, 2018; Mann *et al.*, 2020; Agarwal *et al.*, 2021). Environmental pollution, illicit drug use, smoking, alcohol consumption, dietary exposure to potential endocrine-disrupting chemicals, psychological stress, and unhealthy diets have been hypothesized to be involved in the etiology of poor semen quality (Vander Borght and Wyns, 2018). Given their modifiable nature, decreasing exposure to these could be appropriate in infertility prevention.

Among lifestyle risk factors, dietary habits appear to have an important role in semen quality (Salas-Huetos *et al.*, 2017). Previous research has reported that adherence to healthy dietary patterns rich in unprocessed or minimally processed food (fruits, vegetables, legumes, or nuts) and low in red and processed meat or sugar-sweetened beverages, such as the Mediterranean or Prudent diet is positively associated with semen quality (Afeiche *et al.*, 2014; Salas-Huetos *et al.*, 2019; Benatta *et al.*, 2020; Cao *et al.*, 2022). In contrast, the Western diet, rich in meat and processed meat, dairy products, and sugar-sweetened beverages, has a high glycaemic index and seems to be negatively associated with different semen quality parameters (Nassan *et al.*, 2020). Unfortunately, the Western dietary pattern, which is associated with a higher consumption of ultra-processed food (UPF), has been rising during recent decades (Baker *et al.*, 2020). UPFs are industrial formulations typically of poor nutritional quality and containing several added ingredients including sugar, salt, fat, artificial colors, flavors and stabilizers, among other additives. Thus, they are ready-to-eat, low-cost, hyper-palatable, convenience products with a long shelf life. Additionally, most of them are low in health-beneficial dietary components such as fiber, vitamins, minerals, and phytochemicals (Gibney, 2019; Monteiro *et al.*, 2019). A significant body of scientific evidence has reported an association between UPF consumption and several chronic diseases such as obesity, diabetes, hypertension, cardiovascular disease (CVD), cancer, and all-cause mortality (Chen *et al.*, 2020). To the best of our knowledge, there is only one cross-sectional study exploring the potential relationship between the intake of UPF and semen quality condition. Those findings suggested that higher UPF intake is positively associated with higher odds of asthenozoospermia (Lv *et al.*, 2022). It is worth noting that some studies have focused on the relationship between specific components included in UPF, such as sugar-sweetened and artificially sweetened beverages, and semen quality. Although an inverse relationship between the intake of these components and some sperm parameters has been previously reported (Nassan *et al.*, 2021; Efrat *et al.*, 2022), the findings have been controversial across studies (Meldgaard *et al.*, 2022).

Currently, UPFs constitute a significant and growing component of the global food supply, playing a crucial role in the average consumer’s diet. However, their impact on semen quality has been scarcely studied. Therefore, the aim of this analysis is to further investigate whether UPF consumption in men is associated with semen quality outcomes. We hypothesize that high dietary UPF consumption is negatively associated with the quality of different sperm parameters in men of reproductive age.

## Materials and methods

### Study design and population

A cross-sectional analysis was conducted using data from the first 200 healthy male participants enrolled in the Led-Fertyl (Lifestyle and Environmental Determinants of Seminogram and Other Male Fertility-Related Parameters) study. Healthy male volunteers, aged 18–40 years, from the general population were eligible to participate. The exclusion criteria included severe chronic diseases, reproductive disorders or vasectomy, major organ transplantation, documented CVD, carrier status for HIV or hepatitis B/C infection, acute infections or ongoing inflammation, active cancer or cancer history in the preceding 5 years, severe psychiatric disorders, cirrhosis or liver failure, endocrine diseases, use of antidepressants, calcium channel blockers, alpha-adrenergic blockers, anti-epileptic drugs, anti-retrovirals, immunosuppressive agents or cytotoxic agents, ongoing treatment with systemic corticosteroids, weight loss exceeding 5 kg within the past month, a history of alcohol or drug abuse, or any condition that could potentially impede adherence to the specified study protocol. Participant recruitment took place from February 2021 to April 2023. Several approaches were conducted to enroll potential participants, such as video advertisements in online newspapers and social media, distribution of flyers and posters in various hospitals, primary healthcare centers, pharmacies and stores, dissemination of the study within the university and at public events in the city, among others. Individuals interested in participating subsequently initiated contact with the study staff via phone or email to express their willingness to participate. All participants provided both online and written informed consent.

### Ethical approval

The project’s protocol received approval from the Institut d’Investigació Sanitària Pere i Virgili’s ethical committee (Reference: CEIM: 181/2019) according to the ethical standards laid down in the Declaration of Helsinki.

### Exposure: ultraprocessed food consumption

Participants completed a validated 143-item semi-quantitative food frequency questionnaire in a phone interview with trained dietitians (Fernández-Ballart *et al.*, 2010). Frequency of consumption of each food item, ranging from never or almost never to more than six times per day, during the past year was recorded. Subsequently, responses for each food item were converted into daily grams using the standard portion size of each item. Spanish food composition tables (Mataix Verdú, 2003; Moreiras *et al.*, 2005; Babio *et al.*, 2022) were consulted to compute total daily energy and nutrient intakes.

The NOVA classification system was used to categorize food items based on their degree of processing (Monteiro *et al.*, 2016, 2019). The NOVA system classifies food and beverages into one of four different groups: unprocessed or minimally processed foods (NOVA 1), processed culinary ingredients (NOVA 2), processed foods (NOVA 3), and UPF (NOVA 4). Two independent dietitians executed this classification procedure. Moreover, to ensure meticulous accuracy, further scrutiny by specialists in nutritional epidemiology was performed. Discrepancies in the classification of specific food items were resolved through comprehensive discussion among investigators, leading to final consensus-based decisions.

As the focal point of this study was UPF, the percentage of energy from UPF to total energy intake (% of energy from UPF) was calculated for each participant.

### Outcomes: semen quality parameters

Macroscopic attributes, encompassing semen volume and pH, were evaluated and recorded. Microscopic characteristics were carefully examined using a phase contrast microscope (CX43 Olympus, Tokyo, Japan) in conjunction with a reliable and validated computer-assisted sperm analysis (CASA) system (SCA, Microptic, Barcelona, Spain) (Finelli *et al.*, 2021). This comprehensive assessment comprised parameters including sperm count, sperm concentration, sperm motility, sperm vitality, and sperm morphology.

Participants were provided with explicit instructions to collect semen samples within a sterile standard-polypropylene container via masturbation following a minimum specified abstinence period of 3 days. Subsequent analysis of sperm quality parameters was performed post-liquefaction (for a duration of 30 min at a temperature of 37°C). The procedure of semen collection and its subsequent analysis adhered to the guidelines established by the World Health Organization in 2010 (World Health Organization, 2021).

Specifically, measurements of sperm count and concentration were taken by the CASA system using a 10× phase contrast objective and expressed in terms of millions of sperm per ejaculate or millions of sperm per milliliter, respectively. Sperm motility was analyzed in 200 spermatozoa, appraised by scrutinizing distinct images captured by the CASA system with the 10× phase contrast objective. Each individual spermatozoon was subsequently categorized as progressive motile, non-progressive motile or immotile. The extent of motility was further quantified as a percentage of the total motility, encompassing both progressive and non-progressive motility. The evaluation of sperm vitality involved the hypoosmotic swelling test (HOS test), measured manually with a 60× lens and encompassing an evaluation of 200 spermatozoa. Finally, the assessment of sperm morphology was executed using the Hemacolor (Millipore) staining protocol and 200 spermatozoa were evaluated using the CASA software with the 60× lens.

### Covariate assessment

General lifestyle information (smoking habits and physical activity) and sociodemographic characteristics (age, education level, and income) were collected through online questionnaires. Adherence to a Mediterranean diet was evaluated by a validated 14-item energy-reduced Mediterranean Diet questionnaire in which each item was scored as 1 or 0 points, when the criterion was met or not, respectively (Schröder *et al.*, 2021). Frequency of consumption of extra virgin olive oil, butter, margarine, or cream, vegetables, fruits and juices, meat, fish, legumes, nuts, pastries, caloric and non-caloric artificial sweetened beverages, wine, and Mediterranean tomato sauce (“sofrito”) was collected. The overall score ranged from 0 to 14 points, meaning no-adherence or highest adherence to the Mediterranean diet, respectively. Then, participants underwent in-person assessments at the Hospital Universitari Sant Joan de Reus (Reus, Tarragona, Spain), where anthropometric measurements (weight, height, and waist circumference) and blood pressure were assessed, and biological samples (fasting-blood and semen) were collected. Body mass index (BMI) was calculated as weight in kilograms divided by the square of height in meters.

### Statistical analysis

The statistical analysis employed the latest Led-Fertyl database (May 2023). Normal distribution was assessed by the Kolmogorov–Smirnov test. Total sperm count, sperm concentration, sperm vitality, and normal sperm forms had skewed distributions and were cubic root-transformed to approach normality.

For the baseline characteristics of the study population, continuous variables are reported as mean ± standard deviation (SD) or median (P25, P75) for normal or skewed distributions, respectively. Categorical variables are reported as number (%). To compare differences between groups, one-way analysis of variance (ANOVA) was used for normally distributed variables and the Kruskal–Wallis test was used for variables with a skewed distribution. The Chi-square test was used to comparisons between categorical variables.

Participants were categorized into tertiles of UPF consumption (% of energy from UPF). The first tertile was used as the reference category for all models. UPF consumption was also analyzed as a continuous variable (per 10% increment of energy from UPF consumption to total energy intake). Multivariable linear regression models were fitted to estimate the associations between UPF tertile and semen quality parameters (total sperm count, sperm concentration, sperm vitality, total motility, progressive motility, and normal sperm forms). Results are reported as *β* coefficients and their 95% confidence intervals (CI). All models were adjusted for several potential confounders: Model 1 was adjusted for age (years), education level (primary or secondary education, graduate), and monthly income (less than 1000 €, between 1000 and 2000 € or more than 2000 €). Model 2 (fully adjusted model) was additionally adjusted for sexual abstinence time (days), BMI (kg/m^2^), total energy intake (kcal/day), smoking status (current, former, never), physical activity (tertiles of MET-min/week), and NOVA classification system group excluding group 4 (UPF).

As a sensitivity analysis, the potential effect of the classical dietary quality approach on the association between UPF and semen quality parameters was tested by including the following dietary factors individually in the most adjusted models: alcohol (g/day, tertiles), sodium (mg/day), saturated fatty acids (g/day), fiber (g/day), and fruit and vegetable consumption (g/day). In addition, the main analysis was repeated using non-transformed semen quality parameters as outcomes.

Theoretical mathematical models were used to substitute 10% of energy from unprocessed or minimally processed food consumption with 10% of energy from UPF consumption and test its association with semen quality parameters. The theoretical effect of substituting one food group for another was evaluated by simultaneously adding both variables as continuous variables to the model and the differences in the *β* coefficients, variances and covariance were used to estimate the *β* coefficient and 95% confidence interval (CI) for the substitution effect.

All statistical analyses were performed using Stata/SE software, version 17.0 (StataCorp LP, College Station, TX, USA) and a two-tailed *P* value <0.05 was deemed as statistically significant.

## Results

Among the 320 Led-Fertyl participants assessed for eligibility, 96 were excluded and 24 dropped out of the study. Finally, 200 individuals were included in the current analysis (Supplementary Fig. S1). The average (±SD) age and BMI of these participants was 28.4 ± 5.5 years and 24.4 ± 3.2 kg/m^2^, respectively. The mean (±SD) or the median (IQR) values for the semen parameters were: 48.5 × 10^6^ spz./ml (28.7–83.4) for sperm concentration, 163.5 × 10^6^ spz. (94.6–284.3) for total sperm count, 59.6% (±17.58) for sperm total motility, 43.5% (±17.4) for sperm progressive motility, 9% (5–15) for normal sperm morphology, and 81.5% (75.5—88.5) for sperm vitality. The mean (±SD) of UPF consumption was 236.7 ± 127.5 g/day, which corresponds with 20.64 ± 8.01% energy/day. Table 1 presents the general characteristics of the study population according to tertile of UPF consumption. Participants in the highest tertile were more likely to have a lower total sperm count and lower sperm concentration. Further baseline dietary information across tertiles of UPF consumption is shown in Table 2. Participants with higher consumption of UPF presented a lower intake of protein, total dietary fiber, monounsaturated and polyunsaturated fatty acids, and a higher intake of saturated fatty acids. Individuals consuming more UPF also showed a lower adherence to a Mediterranean diet, a lower consumption of vegetables, fruits, nuts, legumes, and whole cereals and a higher consumption of dairy products, pastry and bakery items, snacks, prepared food, and sauces and seasonings.

**Table 1.** Baseline characteristics of the study participants according to tertiles of ultra-processed food consumption in the Led-Fertyl study.

	UPF consumption (% of energy from UPF)				
		T1	T2	T3	
	n	n = 67	n = 67	n = 66	*P*-value
UPF consumption, % energy	200	12.2 ± 3.4	20.4 ± 1.8	29.5 ± 5.3	<0.001
UPF consumption, NOVA 4, g/day	200	141.9 ± 73.6	227.4 ± 86.5	342.3 ± 126.3	<0.001
Processed foods consumption, NOVA 3, g/day	200	35.0 [25.7–55.0]	49.9 [26.4–60.0]	33.6 [25.7–50.0]	0.059
Processed culinary ingredients consumption, NOVA 2, g/day	200	390.7 [217.0–480.8]	372.4 [241.3–502.9]	308.7 [223.3–471.1]	0.432
Unprocessed and minimally processed foods consumption, NOVA 1, g/day	200	1322.3 ± 455.5	1142.8 ± 379.8	1017.5 ± 274.4	<0.001
**Demographic characteristics**					
Age (years)	200	28.2 ± 6.1	28.8 ± 5.7	28.2 ± 4.8	0.754
BMI (kg/m^2^)	200	24.0 ± 2.6	24.4 ± 3.1	24.8 ± 3.8	0.430
Waist circumference (cm)	200	81 [77–83.6]	82 [77.3–87.8]	84.6 [76.7–90.5]	0.167
Systolic blood pressure (mmHg)	200	127.2 ± 10.2	127.7 ± 9.94	128.1 ± 10.8	0.881
Diastolic blood pressure (mmHg)	200	73.5 ± 8.9	73.2 ± 7.6	75.1 ± 10.9	0.461
Physical activity (MET/h/week)	200	4065 [2239–6182]	3282 [1681–4918]	3142 [1795–4774]	0.083
Abstinence time (days)	200	3.5 [2.4–4.2]	3.5 [3–4.7]	3.3 [2.5–4.5]	0.311
Educational level (n, %)	200				0.499
High school or less		21 (31.3)	23 (34.3)	27 (40.9)	
College or high education		46 (68.6)	44 (65.7)	39 (59.1)	
Monthly income (n, %)	167				0.009
Less than 1000 €		15 (22.4)	6.0 (5.97)	12 (18.2)	
Between 1000 € and 2000 €		40 (59.7)	43 (64.2)	47 (71.2)	
More than 2000 €		12 (17.9)	20 (29.9)	7 (10.6)	
Smoking, n (%)	188				0.142
Never		56 (83.6)	49 (73.1)	44 (66.7)	
Current		4 (6.0)	8 (11.9)	13 (19.7)	
Former		7 (10.5)	10 (14.9)	9 (13.6)	
Civil status (n, %)	200				0.232
Single		49 (73.1)	49 (73.1)	56 (84.9)	
Married		6 (9.0)	10 (14.9)	4 (6.1)	
Other		12 (17.9)	8 (11.9)	6 (9.1)	
**Semen parameters**					
pH	200	8.5 [8–8.5]	8.5 [8–8.5]	8.5 [8.5–8.5]	0.124
Semen volume (ml)	200	3.5 [2.4–4.2]	3.5 [3–4.7]	3.3 [2.5–4.5]	0.311
Sperm concentration (×10^6^/ml)	200	55.5 [31.32–85.6]	51.08 [33.18–91.5]	41.5 [20.9–65.7]	0.037
Total sperm count (×10^6^)	200	158.5 [104.5–286.3]	213.1 [126.8–332.5]	127.0 [72.3–245.4]	0.015
Total motility (%)	200	61.2 ± 16.7	61.2 ± 15.8	56.3 ± 19.5	0.175
Progressive motility (%)	200	44.41 ± 18.0	45.1 ± 16.3	40.8 ± 17.7	0.312
Normal sperm morphology (%)	199	10 [5–15]	8.5 [4.5–17]	8 [4.5–14]	0.587
Sperm vitality (%)	199	85 [79–91]	80 [72.5–85]	81 [74–87.5]	0.014
**Blood parameters**					
Plasma glucose (mg/dl)	199	88.7 ± 7.6	89.1 ± 6.9	89.1 ± 6.4	0.931
Total cholesterol (mg/dl)	200	174.0 ± 26.8	173.9 ± 28.9	173.4 ± 28.5	0.990
HDL-c (mg/dl)	197	57.9 ± 13.0	56.9 ± 11.6	54.4 ± 11.6	0.226
LDL-c (mg/dl)	197	99.4 ± 26.6	99.4 ± 27.3	100.0 ± 25.1	0.989

BMI, body mass index; METS, metabolic equivalent of task; T, tertile; UPF, ultra-processed food. Values are reported as means ± standard deviations or median (Pc25–Pc75) for continuous variables and number (%) for categorical variables. *P*-value was calculated by one-way analysis of variance (ANOVA) test or Kruskal–Wallis test for continuous normal or non-normal distributed variables, respectively. Chi-square test was used to compare categorical variables.

**Table 2:** Dietary baseline characteristics across tertiles of baseline ultra-processed food consumption of the study population.

	UPF consumption (% of energy from UPF)			
	T1	T2	T3	
	n = 67	n = 67	n = 66	*P*-value
**Energy (kcal/day)**	2589 ± 716	2678 ± 619	2676 ± 561	0.654
**Nutrients**				
Proteins (% energy)	17.0 ± 2.4	16.5 ± 2.56	15.3 ± 2.0	<0.001
Proteins (g/day)	109.5 ± 32.5	110.0 ± 30.1	102.0 ± 25.7	0.222
Fats (% energy)	41.4 ± 6.9	43.5 ± 6.0	42.6 ± 5.1	0.133
Fats (g/day)	118.2 ± 37.1	128.9 ± 32.9	126.7 ± 31.1	0.155
Saturated FA (% of total fats)	25.7 ± 4.6	28.3 ± 4.4	29.7 ± 3.8	<0.001
Monounsaturated FA (% of total fats)	47.7 ± 4.7	46.4 ± 4.6	45.1 ± 3.9	0.004
Polyunsaturated FA (% of total fats)	14.6 [13.0–17.7]	13.1 [11.5–14.4]	13.1 [11.6–14.6]	<0.001
Carbohydrates (% energy)	39.5 ± 7.3	37.7 ± 6.5	39.3 ± 5.7	0.244
Carbohydrates (g/day)	257.9 ± 94.2	254.4 ± 78.7	262.9 ± 66.5	0.828
Total dietary fiber (g/day)	27.0 [20.1–33.4]	22.7 [16.5–28.6]	21.4 [16.5–25.1]	<0.001
Sodium (mg/day)	2493.1 [1878.6–3011.4]	2822.0 [2259.1–3507.2]	2666.2 [2322.4–3425.6]	0.007
**Mediterranean diet adherence, points**	9 [8–10]	8 [7–9]	7 [6–8]	<0.001
**Food subgroups, g/day**				
Dairy	235.2 [94.1–334.5]	260.7 [145.0–328.6]	205.9 [106.9–274.8]	0.070
Dairy products	13.3 [0–25.2]	15.3 [6.7–42.9]	25.4 [15.3–45.2]	<0.001
Eggs	47.1 [25.7–47.1]	25.7 [25.7–47.1]	25.7 [25.7–47.1]	0.450
Meat and meat products	150.2 ± 83.8	182.2 ± 85.5	172.6 ± 89.4	0.090
Fish and seafood	85.7 ± 47.9	90.8 ± 48.1	78.6 ± 42.1	0.311
Vegetables	303.3 [217.1–385.3]	213.2 [168.4–283.2]	197.0 [149.3–258.6]	<0.001
Tubers	85.7 [34.7–150.0]	85.7 [23.3–95.7]	50 [23.3–95.7]	0.074
Fruits	207.1 [149.7–296.4]	153.3 [82.9–256.9]	157.7 [110.7–221.4]	0.005
Nuts	25.7 [8.6–38.6]	17.1 [8–32.0]	11.4 [4.3–21.4]	0.002
Legumes	29.7 [17.1–51.4]	16.6 [12–29.7]	21.1 [12.6–33.7]	<0.001
Cereals	83.6 [40.1–126.4]	109 [68.4–217.2]	88.4 [68.9–126.4]	0.067
Whole cereals	35 [9.3–83.3]	10.7 [0–45.0]	12.9 [0–38.4]	<0.001
Oils and fats	27.9 [25–51.3]	33.6 [25.0–51.3]	27.9 [25.0–49.3]	0.440
Pastry and bakery	15.3 [8.3–32.5]	32.4 [19.1–49.4]	50.7 [31.4–81.0]	<0.001
Sugar and sweetened	5.0 [0.7–12.8]	10.0 [2.9–16.4]	7.8 [1.3–15.0]	0.175
Snack	6.7 [3.3–10.5]	10.5 [6.7–14.3]	12.4 [6.7–24.8]	<0.001
Prepared food	16 [13.3–30.6]	28.6 [15.3–32.9]	85.7 [32.9–87.7]	<0.001
Sauces and seasonings	1.4 [0–4.3]	4.3 [0.7–7.9]	4.3 [1.4–7.9]	<0.001
Sweetened beverages	41.9 [26.7–171.4]	85.7 [26.7–170.5]	112.4 [41.9–200.0]	0.139
Alcoholic beverages	144.8 [32–172.4]	154.8 [22–233.8]	144.8 [52.9–230.0]	0.514
Coffee and tea	60.7 [28.6–125.0]	53.3 [21.4–125.0]	50.0 [10.5–125.0]	0.227

FA, fatty acids; T, tertile; UPF, ultra-processed food. Values are reported as means ± standard deviations or median (Pc25–Pc75). *P*-value was calculated by one-way analysis of variance (ANOVA) test or Kruskal–Wallis test for normal or non-normal distributed variables, respectively.

The cross-sectional associations (*β* coefficient; 95% CI) between tertiles of UPF consumption and semen quality parameters are displayed in Table 3. Across tertiles of UPF consumption, in the fully adjusted model, UPF consumption showed a statistically significant inverse association with sperm concentration (*β*: −1.42 × 10^6^ spz./ml; 95% CI: −2.72 to −0.12) and total motility (*β*: −7.83%; 95% CI: −15.16 to −0.51). When UPF was analyzed as a continuous variable, each 10% increment of energy from UPF was inversely associated with total sperm count in all of the models. In the fully adjusted model, each 10% of energy from UPF consumption increase was associated with a −1.50 × 10^6^ spz. decrease in total sperm count (95% CI: −2.83 to −0.17). In general, these main results did not change substantially when sensitivity analyses were performed by including specific dietary factors individually in the most adjusted models or when non-transformed semen quality parameter variables were used as main outcomes (Supplementary Tables S1 and S2).

**Table 3:** Multivariable-adjusted *β* coefficients and 95% CI of semen quality parameters across tertiles and per 10% increment of ultra-processed food consumption.

	UPF consumption (% of energy from UPF)				Per 10% increment
	T1	T2	T3	*P*-trend	(n = 200)
	(n = 67)	(n = 67)	(n = 66)		
UPF consumption, % energy	[3.26–17.14]	[17.35–23.16]	[23.29–46.14]		[3.26–46.14]
**Total sperm count (×10^6^ spz.)^a^**					
Crude model	0 (Ref.)	0.82 (−1.13 to 2.77)	−1.74 (−3.70 to 0.22)	0.097	−**1.12 (**−**2.12 to** −**0.12)**
Model 1	0 (Ref.)	0.75 (−1.24 to 2.75)	−1.79 (−3.76 to 0.18)	0.089	−**1.12 (**−**2.13 to** −**0.11)**
Model 2	0 (Ref.)	0.57 (−1.53 to 2.68)	−2.20 (−4.58 to 0.19)	0.090	−**1.50 (**−**2.83 to** −**0.17)**
**Sperm concentration (×10^6^ spz./ml)^a^**					
Crude model	0 (Ref.)	−0.03 (−1.08 to 1.02)	−**1.08 (**−**2.14 to** −**0.03)**	0.048	−0.49 (−1.03 to 0.05)
Model 1	0 (Ref.)	−0.11 (−1.19 to 0.97)	−**1.08 (**−**2.14 to** −**0.01)**	0.051	−0.48 (−1.02 to 0.07)
Model 2	0 (Ref.)	−0.26 (−1.41 to 0.89)	−**1.42 (**−**2.72 to** −**0.12)**	0.037	−0.66 (−1.38 to 0.07)
**Sperm vitality (%)^a^**					
Crude model	0 (Ref.)	−0.15 (−0.53 to 0.24)	−0.27 (−0.65 to 0.12)	0.166	−0.05 (−0.25 to 0.15)
Model 1	0 (Ref.)	−0.23 (−0.61 to 0.16)	−0.30 (−0.68 to 0.09)	0.127	−0.07 (−0.27 to 0.12)
Model 2	0 (Ref.)	−0.26 (−0.67 to 0.15)	−0.45 (−0.92 to 0.01)	0.055	−0.14 (−0.40 to 0.12)
**Total motility (%)**					
Crude model	0 (Ref.)	0.001 (−5.93 to 5.93)	−4.91 (−10.86 to 1.04)	0.113	−1.19 (−4.24 to 1.86)
Model 1	0 (Ref.)	0.42 (−5.66 to 6.50)	−5.07 (−11.08 to 0.94)	0.107	−1.23 (−4.32 to 1.87)
Model 2	0 (Ref.)	−1.01 (−7.47 to 5.45)	−**7.83 (**−**15.16 to** −**0.51)**	0.042	−2.48 (−6.58 to 1.63)
**Progressive motility (%)**					
Crude model	0 (Ref.)	0.69 (−5.21 to 6.60)	−3.60 (−9.52 to 2.33)	0.246	−0.56 (−3.59 to 2.47)
Model 1	0 (Ref.)	1.20 (−4.88 to 7.28)	−3.89 (−9.90 to 2.12)	0.220	−0.65 (−3.74 to 2.44)
Model 2	0 (Ref.)	−0.48 (−6.98 to 6.01)	−7.33 (−14.70 to 0.03)	0.059	−2.25 (−6.38 to 1.87)
**Normal sperm forms (%)^a^**					
Crude model	0 (Ref.)	−0.02 (−0.43 to 0.38)	−0.21 (−0.62 to 0.19)	0.307	−0.14 (−0.40 to 0.12)
Model 1	0 (Ref.)	−0.03 (−0.45 to 0.39)	−0.21 (−0.63 to 0.20)	0.311	−0.14 (−0.35 to 0.07)
Model 2	0 (Ref.)	0.004 (−0.44 to 0.45)	−0.23 (−0.74 to 0.27)	0.378	−0.20 (−0.47 to 0.08)

UPF, ultra-processed food; T, tertiles; Ref, reference. Model 1 was adjusted by age (years), education level (primary and secondary education, graduate) and income (less than 1000 EU, between 1000 and 2000 EU and more than 2000 EU). Model 2 was additionally adjusted by abstinence time (days), body mass index (kg/m^2^), total energy intake (kcal/day), smoking status (current, former, never), physical activity (tertiles of MET-min/week), and NOVA classification system groups except group 4.

a Total sperm count, sperm concentration, sperm vitality, and normal sperm forms were cubic root-transformed to more closely approximate to a normal distribution. Bold indicates *P*-value < 0.05.

Table 4 presents the theoretical replacement of 10% of energy from UPF consumption with 10% of energy from unprocessed or minimally processed food consumption. This was associated with increases of 1.78 × 10^6^ spz. (95% CI: 0.29 to 3.27) in total sperm count, 0.89 × 10^6^ spz./ml (95% CI: 0.07 to 1.70) in sperm concentration, 5.80% (95% CI: 1.27 to 10.34) in total motility, 5.76% (95% CI: 1.22 to 10.30) in progressive motility, and 0.32% (95% CI: 0.01 to 0.64) in normal sperm forms, after adjusting for potential confounders. The results of this substitution analysis were not significant in the case of sperm vitality.

**Table 4:** β-coefficients and 95% CI of semen quality parameters for substituting 10% of energy from unprocessed or minimally processed food consumption for 10% of energy from ultra-processed food consumption.

	Substitution of UMPF consumption for UPF consumption
	(n = 200)
**Total sperm count (×10^6^ spz.)^a^**	
Fully adjusted model	**1.78 (0.29 to 3.27)**
**Sperm concentration (×10^6^ spz./ml)^a^**	
Fully adjusted model	**0.89 (0.07 to 1.70)**
**Sperm vitality (%)^a^**	
Fully adjusted model	0.09 (−0.21 to 0.38)
**Total motility (%)**	
Fully adjusted model	**5.80 (1.27 to 10.34)**
**Progressive motility (%)**	
Fully adjusted model	**5.76 (1.22 to 10.30)**
**Normal sperm forms (%)^a^**	
Fully adjusted model	**0.32 (0.01 to 0.64)**

UMPF, unprocessed or minimally processed food; UPF, ultra-processed food. Values are based in the 10% of energy from specific group food consumption. Linear regression models were adjusted by age (years), education level (primary and secondary education, graduate), income (less than 1000 EU, between 1000 and 2000 EU and more than 2000 EU), abstinence time (days), body mass index (kg/m^2^), total energy intake (kcal/day), smoking status (current, former, never), physical activity (tertiles of MET-min/week), and NOVA classification system groups 2 and 3.

a Total sperm count, sperm concentration, sperm vitality, and normal sperm forms were cubic root-transformed to more closely approximate a normal distribution. Bold indicates *P*-value < 0.05.

## Discussion

To the best of our knowledge, this is the first study using the NOVA classification system to examine the association between UPF consumption and several semen quality parameters. The findings of this cross-sectional analysis conducted in young healthy men suggest that higher consumption of UPF is associated with lower total sperm count, concentration and total motility. Moreover, replacing 10% of energy from UPF consumption with unprocessed or minimally processed food was associated with increases in total sperm count, sperm concentration, total motility, progressive motility, and normal sperm forms.

Epidemiological evidence regarding the potential relationship between UPF consumption and semen quality is extremely limited. In fact, as far as we know, only one case-control study has investigated the association between intake of UPF and asthenozoospermia. That study conducted in a population of 1130 Chinese young adult men reported higher odds of asthenozoospermia in those with high UPF intake (Lv *et al.*, 2022). Our results not only support these previous findings with regard to sperm vitality, but also add further evidence by showing significant associations with other semen quality parameters such as sperm count and concentration. Nevertheless, it is worth mentioning that, although the described inverse associations between UPF consumption and semen quality parameters were statistically significant, whether or not this translates into clinical effects on fertility outcomes deserves further research.

UPF consumption is one of the main characteristics of the Western diet (Clemente-Suárez *et al.*, 2023). While research regarding UPF and semen quality is scarce, to date, evidence regarding dietary patterns aligned with UPF consumption is more abundant and indicates that Western diet adherence is associated with poor semen quality (Liu *et al.*, 2015; Danielewicz *et al.*, 2018) and a higher risk of asthenozoospermia (Eslamian *et al.*, 2016). In fact, high consumption of some specific food groups, considered UPFs and typical of the Western diet, such as processed meat or sugar-sweetened beverages, was reported to be negatively associated with normal sperm morphology or sperm motility (Afeiche *et al.*, 2014; Chiu *et al.*, 2014). However, other authors have not found such a significant association between the Western diet and semen quality (Gaskins *et al.*, 2012; Jurewicz *et al.*, 2018)*.* Discrepancies between these studies may be due to differences in dietary assessment methods or the methodology used to construct Western dietary patterns. Therefore, the potential role of UPF consumption on semen quality parameters, as an indicator of male infertility, should be further investigated in future studies.

The potential biological mechanisms linking UPF consumption to worsening semen quality parameters are not entirely clear. Overall, major characteristics of UPF include their high energy density, high simple sugar, trans-fatty acids, and sodium contents as well as their lower fiber content and antioxidant micronutrients, resulting in substantially low nutritional quality compared to unprocessed or minimally processed food (Gibney, 2019; Monteiro *et al.*, 2019; Whittaker, 2023). High trans-fatty acid intake has been previously linked to lower semen quality (Attaman *et al.*, 2012; Chavarro *et al.*, 2014; Ricci *et al.*, 2020) and higher odds of asthenozoospermia (Eslamian *et al.*, 2016) in young men. Sugar intake has also been suggested as a contributor to semen quality decline, especially when derived from sugar-sweetened beverages. Previous epidemiologic studies have reported its negative associations with sperm concentration (Efrat *et al.*, 2022), count (Nassan *et al.*, 2021), and motility (Chiu *et al.*, 2014). High UPF consumers in our study had lower intake of the principal dietary sources of antioxidants such as vegetables, fruits, nuts, and legumes. A previous case-control study in Mediterranean young men reported that lower dietary intake of antioxidants was related to poor semen quality (Mendiola *et al.*, 2010). Several specific antioxidants such as vitamins C, E, β-carotene, folate, zinc, and omega-3 have also been linked to sperm quality parameters by preceding studies (Keskes-Ammar *et al.*, 2003; Eskenazi *et al.*, 2005; Akmal *et al.*, 2006; Young *et al.*, 2008; Attaman *et al.*, 2012; Mínguez-Alarcón *et al.*, 2012; Falsig *et al.*, 2019). Besides, these compounds may have an indirect protective effect on semen quality by avoiding reactive oxygen species production and oxidative stress (Bisht *et al.*, 2017). It is worth mentioning that in our study, further adjustment for the aforementioned key nutrients or fruit and vegetable consumption attenuated the association between UPF consumption and semen quality parameters, but did not alter the sense or significance of the results. Therefore, other potential factors (i.e. exposure to endocrine disruptors, additive combinations or changes in nutrient availability caused by the food matrix) need to be investigated in the future. The production of convenience ready-to-eat UPF products itself facilitates the transfer of certain endocrine-disrupting chemical compounds (i.e. bisphenols, phthalates) from the plastic packaging to the food, along with the intake of food preservatives (i.e. parabens), which have been associated with lower semen quality and DNA damage (Virant-Klun *et al.*, 2022; Whittaker, 2023). However, a recent systematic review and meta-analysis reported a disparity between studies exploring associations between endocrine-disrupting chemicals and semen quality, and although the authors claimed that further research was needed, they also recommended to minimize exposure to these compounds as much as possible (Martínez *et al.*, 2023).

This study has some limitations that should be considered when interpreting the results. First, the cross-sectional design nature of this study does not allow inferences about causation to be drawn. Second, this study was conducted in healthy young Mediterranean men and consequently, the findings cannot be extrapolated to other populations. UPF consumption was estimated from a food frequency questionnaire not specifically designed for assessing this type of food, so some misclassification might have occurred in the case of additives or preparation methods not covered by our questionnaire. However, the NOVA classification system has been widely used in previous epidemiological studies as an easy-to-apply and suitable method for food processing classification facilitating comparison between studies and provision of simple advice to the general population. Food frequency questionnaires are prone to possible measurement errors and reporting bias but are widely used in nutritional studies and ours was carefully administered by trained dietitians and thorough checks for implausible total energy intake were applied.

Notable strengths of the study include the novelty of the study that, to the best of our knowledge, reports an association between UPF consumption and several semen quality parameters, for the first time, in young and healthy men. A strict protocol was used for sample handling, processing and analysis using the CASA system, and by the same researcher. Furthermore, the extensive sociodemographic and lifestyle data collected provided a wide selection of covariates for the control of potential bias in the models applied. However, possible residual confounding bias cannot entirely be ruled out.

## Conclusion

High dietary UPF consumption was inversely associated with certain semen quality parameters, including total sperm count, sperm concentration, and total motility, in a population of young and healthy men. Additionally, our results suggest that unprocessed and minimally processed food consumption instead of UPF could have a beneficial effect on semen quality parameters. Although the observed results could help to update or even develop preventive and interventional male infertility programs, further studies are required to replicate our observations, extend them to other populations, and examine the underlying biological mechanisms explaining the associations found, specifically long-term and/or well-controlled clinical trials.

## Supplementary Material

hoae001_Supplementary_Data

## Data Availability

The datasets produced and examined in the course of this present study are not accessible to the public, owing to data regulations and ethical considerations. This precaution has been taken to safeguard the consent of research participants, as their original agreement was limited to the utilization of their data by the initial research team. Nevertheless, the possibility of collaborative data analysis can be explored by submitting a formal request via a letter addressed to the corresponding author (nancy.babio@urv.cat).
