# Supplementary material for: Ultra-processed food consumption and semen quality parameters in the Led-Fertyl study
Source: Hum Reprod Open. 2024 Jan 17;2024(1):hoae001. doi: 10.1093/hropen/hoae001 (PMC10813743; doi:10.1093/hropen/hoae001)
Supplement: hoae001_Supplementary_Data [file hoae001_supplementary_data.zip › HRO-23-0280-R2-SuppFig1_2.docx]

**Supplementary Figure S1.** Flow diagram for the study population.
