# Supplementary material for: Ultra-processed food consumption and semen quality parameters in the Led-Fertyl study
Source: Hum Reprod Open. 2024 Jan 17;2024(1):hoae001. doi: 10.1093/hropen/hoae001 (PMC10813743; doi:10.1093/hropen/hoae001)
Supplement: hoae001_Supplementary_Data [file hoae001_supplementary_data.zip › Supplementary_Tables.docx]

**Supplementary Table S1.**  **Sensitivity analysis of the association between ultra-processed food consumption and semen quality parameters including specific dietary factors**

|  | **UPF consumption (% of energy from UPF)** | | | | **Per 10% increment** |
| --- | --- | --- | --- | --- | --- |
|  | **T1**  **(n=67)** | **T2**  **(n=67)** | **T3**  **(n=66)** | ***P*-trend** | **(n=200)** |
| **Total sperm count (x10^6^spz.)^a^** |  |  |  |  |  |
| + alcohol intake (tertiles of g/d) | 0 (Ref.) | 0.62 (-1.50 to 2.73) | -2.12 (-4.54 to 0.29) | 0.110 | **-1.46 (-2.81 to -0.11)** |
| + sodium intake (mg/d) | 0 (Ref.) | 0.53 (-1.63 to 2.68) | -2.22 (-4.61 to 0.18) | 0.083 | **-1.50 (-2.83 to -0.17)** |
| + saturated fatty acids intake (g/d) | 0 (Ref.) | 0.87 (-1.24 to 2.99) | -1.92 (-4.31 to 0.47) | 0.139 | -1.29 (-2.65 to 0.07) |
| + fiber intake (g/d) | 0 (Ref.) | 1.29 (-0.90 to 3.48) | -1.50 (-3.95 to 0.96) | 0.238 | -1.14 (-2.56 to 0.29) |
| + vegetable and fruit consumption (g/d) | 0 (Ref.) | 0.76 (-1.39 to 2.91) | -2.10 (-4.50 to 0.30) | 0.102 | **-1.47 (-2.81 to -0.13)** |
| **Sperm concentration (x10^6^spz./ml)^a^** |  |  |  |  |  |
| + alcohol intake (tertiles of g/d) | 0 (Ref.) | -0.23 (-1.39 to 0.93) | **-1.34 (-2.66 to -0.03)** | 0.052 | -0.61 (-1.35 to 0.13) |
| + sodium intake (mg/d) | 0 (Ref.) | -0.30 (-1.48 to 0.88) | **-1.44 (-2.75 to -0.13)** | 0.034 | -0.66 (-1.39 to 0.07) |
| + saturated fatty acids intake (g/d) | 0 (Ref.) | -0.16 (-1.33 to 1.00) | **-1.33 (-2.64 to -0.02)** | 0.052 | -0.59 (-1.33 to 0.16) |
| + fiber intake (g/d) | 0 (Ref.) | -0.09 (-1.29 to 1.13) | -1.24 (-2.60 to 0.11) | 0.072 | -0.56 (-1.34 to 0.23) |
| + vegetable and fruit consumption (g/d) | 0 (Ref.) | -0.23 (-1.40 to 0.95) | **-1.40 (-2.72 to -0.09)** | 0.040 | -0.65 (-1.39 to 0.08) |
| **Sperm vitality (%)^a^** |  |  |  |  |  |
| + alcohol intake (tertiles of g/d) | 0 (Ref.) | -0.26 (-0.67 to 0.16) | -0.47 (-0.94 to 0.004) | 0.072 | -0.16 (-0.42 to 0.11) |
| + sodium intake (mg/d) | 0 (Ref.) | -0.24 (-0.67 to 0.18) | -0.45 (-0.92 to 0.02) | 0.061 | -0.14 (-0.40 to 0.12) |
| + saturated fatty acids intake (g/d) | 0 (Ref.) | -0.30 (-0.72 to 0.12) | **-0.49 (-0.96 to -0.02)** | 0.040 | -0.18 (-0.45 to 0.09) |
| + fiber intake (g/d) | 0 (Ref.) | -0.27 (-0.71 to 0.16) | -0.47 (-0.95 to 0.02) | 0.060 | -0.15 (-0.43 to 0.14) |
| + vegetable and fruit consumption (g/d) | 0 (Ref.) | -0.26 (-0.68 to 0.16) | -0.45 (-0.93 to 0.02) | 0.058 | -0.14 (-0.41 to 0.12) |
| **Total motility (%)** |  |  |  |  |  |
| + alcohol intake (tertiles of g/d) | 0 (Ref.) | -0.78 (-7.28 to 5.72) | **-7.78 (-15.19 to -0.36)** | 0.047 | -2.48 (-6.65 to 1.70) |
| + sodium intake (mg/d) | 0 (Ref.) | -1.88 (-8.49 to 4.72) | **-8.16 (-15.49 to -0.82)** | 0.032 | -2.50 (-6.60 to 1.60) |
| + saturated fatty acids intake (g/d) | 0 (Ref.) | -1.25 (-7.80 to 5.30) | **-8.05 (-15.45 to -0.65)** | 0.037 | -2.76 (-6.97 to 1.46) |
| + fiber intake (g/d) | 0 (Ref.) | -0.97 (-7.77 to 5.83) | **-7.79 (-15.41 to -0.16)** | 0.046 | -2.51 (-6.93 to 1.91) |
| + vegetable and fruit consumption (g/d) | 0 (Ref.) | -1.50 (-8.10 to 5.11) | **-8.09 (-15.46 to -0.72)** | 0.034 | -2.72 (-6.87 to 1.43) |
| **Progressive motility (%)** |  |  |  |  |  |
| + alcohol intake (tertiles of g/d) | 0 (Ref.) | -0.25 (-6.79 to 6.29) | -7.14 (-14.59 to 0.32) | 0.050 | -2.14 (-6.34 to 2.06) |
| + sodium intake (mg/d) | 0 (Ref.) | -2.14 (-8.71 to 4.44) | **-7.95 (-15.25 to -0.65)** | 0.035 | -2.29 (-6.37 to 1.78) |
| + saturated fatty acids intake (g/d) | 0 (Ref.) | -0.40 (-6.99 to 6.19) | -7.26 (-14.70 to 0.19) | 0.063 | -2.23 (-6.47 to 2.02) |
| + fiber intake (g/d) | 0 (Ref.) | -0.59 (-7.43 to 6.25) | -7.44 (-15.11 to 0.23) | 0.058 | -2.42 (-6.86 to 2.03) |
| + vegetable and fruit consumption (g/d) | 0 (Ref.) | -1.03 (-7.67 to 5.62) | **-7.63 (-15.04 to -0.22)** | 0.048 | -2.52 (-6.69 to 1.64) |
| **Normal sperm forms (%)^a^** |  |  |  |  |  |
| + alcohol intake (tertiles of g/d) | 0 (Ref.) | 0.03 (-0.41 to 0.47) | -0.18 (-0.69 to 0.32) | 0.500 | -0.17 (-0.45 to 0.11) |
| + sodium intake (mg/d) | 0 (Ref.) | -0.13 (-0.58 to 0.31) | -0.28 (-0.78 to 0.21) | 0.259 | -0.20 (-0.47 to 0.08) |
| + saturated fatty acids intake (g/d) | 0 (Ref.) | 0.004 (-0.45 to 0.45) | -0.23 (-0.74 to 0.27) | 0.379 | -0.20 (-0.49 to 0.08) |
| + fiber intake (g/d) | 0 (Ref.) | -0.06 (-0.52 to 0.41) | -0.30 (-0.81 to 0.23) | 0.266 | -0.27 (-0.56 to 0.03) |
| + vegetable and fruit consumption (g/d) | 0 (Ref.) | -0.04 (-0.49 to 0.41) | -0.26 (-0.76 to 0.25) | 0.323 | -0.22 (-0.50 to 0.06) |
| **Abbreviations:** UPF, Ultra-processed food; T, Tertiles; *Ref,* Reference.  Linear regression models were adjusted by age (years), education level (primary and secondary education, graduate), income (less than 1000 EU, between 1000-2000 EU and more than 2000 EU), abstinence time (days), body mass index (kg/m2), total energy intake (kcal/day), smoking status (current, former, never), physical activity (tertiles of MET-min/week), NOVA classification system groups except group 4 and variables indicated in each row.  **^a^**Total sperm count, sperm concentration, sperm vitality and normal sperm forms were cubic root-transformed to more closely approximate a normal distribution.  Bold indicates p-value<0.05. | | | | | |

**Supplementary Table S2.**  **Multivariable-adjusted β-coefficients and 95% CI of semen quality parameters across tertiles and per 10% increment of ultra-processed food consumption**

|  | **UPF consumption (% of energy from UPF)** | | | | **Per 10% increment** |
| --- | --- | --- | --- | --- | --- |
|  | **T1**  **(n=67)** | **T2**  **(n=67)** | **T3**  **(n=66)** | ***P*-trend** | **(n=200)** |
| UPF consumption, % energy | [3.26-17.14] | [17.35-23.16] | [23.29-46.14] |  | [3.26-46.14] |
| **Total sperm count (x10^6^spz.)** |  |  |  |  |  |
| Crude model | 0 (Ref.) | 14.88 (-46.08 to 75.83) | -51.69 (-112.87 to 9.50) | 0.079 | **-39.80 (-72.91 to -6.69)** |
| Model 1 | 0 (Ref.) | 15.28 (-46.32 to 76.88) | -52.85 (-113.71 to 8.01) | 0.113 | **-31.12 (-61.49 to -0.75)** |
| Model 2 | 0 (Ref.) | 10.67 (-54.77 to 76.11) | -61.71 (-135.91 to 12.50) | 0.096 | **-44.91 (-84.98 to -4.84)** |
| **Sperm concentration (x10^6^spz./ml)** |  |  |  |  |  |
| Crude model | 0 (Ref.) | -2.22 (-20.11 to 15.66) | -16.27 (-34.22 to 1.68) | 0.055 | -9.32 (-19.05 to 0.41) |
| Model 1 | 0 (Ref.) | -2.94 (-21.22 to 15.34) | -15.87 (-33.93 to 2.19) | 0.080 | -7.61 (-16.78 to 1.56) |
| Model 2 | 0 (Ref.) | -5.05 (-24.66 to 14.56) | -19.27 (-41.51 to 2.97) | 0.069 | -10.34 (-22.38 to 1.70) |
| **Sperm vitality (%)** |  |  |  |  |  |
| Crude model | 0 (Ref.) | -3.77 (-8.48 to 0.94) | -4.46 (-9.21 to 0.29) | 0.082 | -0.94 (-3.49 to 1.60) |
| Model 1 | 0 (Ref.) | -4.14 (-8.97 to 0.68) | -4.51 (-9.30 to 0.28) | 0.072 | -1.03 (-3.47 to 1.42) |
| Model 2 | 0 (Ref.) | -4.15 (-9.27 to 0.97) | **-5.93 (-11.76 to -0.11)** | 0.055 | -1.40 (-4.56 to 1.77) |
| **Total motility (%)** |  |  |  |  |  |
| Crude model | 0 (Ref.) | 0.001 (-5.93 to 5.93) | -4.91 (-10.86 to 1.04) | 0.113 | -1.19 (-4.24 to 1.86) |
| Model 1 | 0 (Ref.) | 0.42 (-5.66 to 6.50) | -5.07 (-11.08 to 0.94) | 0.107 | -1.23 (-4.32 to 1.87) |
| Model 2 | 0 (Ref.) | -1.01 (-7.47 to 5.45) | **-7.83 (-15.16 to -0.51)** | 0.042 | -2.48 (-6.58 to 1.63) |
| **Progressive motility (%)** |  |  |  |  |  |
| Crude model | 0 (Ref.) | 0.69 (-5.21 to 6.60) | -3.60 (-9.52 to 2.33) | 0.246 | -0.56 (-3.59 to 2.47) |
| Model 1 | 0 (Ref.) | 1.20 (-4.88 to 7.28) | -3.89 (-9.90 to 2.12) | 0.220 | -0.65 (-3.74 to 2.44) |
| Model 2 | 0 (Ref.) | -0.48 (-6.98 to 6.01) | -7.33 (-14.70 to 0.03) | 0.059 | -2.25 (-6.38 to 1.87) |
| **Normal sperm forms (%)** |  |  |  |  |  |
| Crude model | 0 (Ref.) | -0.07 (-2.58 to 2.44) | -0.97 (-3.48 to 1.54) | 0.607 | -0.81 (-2.15 to 0.54) |
| Model 1 | 0 (Ref.) | -0.04 (-2.62 to 2.55) | -0.93 (-3.49 to 1.62) | 0.403 | -0.98 (-2.26 to 0.31) |
| Model 2 | 0 (Ref.) | 0.25 (-2.48 to 2.99) | -0.79 (-3.89 to 2.30) | 0.575 | -1.16 (-2.83 to 0.50) |
| **Abbreviations:** UPF, Ultra-processed food; T, Tertiles; *Ref,* Reference.  Model 1 was adjusted by age (years), education level (primary and secondary education, graduate) and income (less than 1000 EU, between 1000-2000 EU and more than 2000 EU). Model 2 was additionally adjusted by abstinence time (days), body mass index (kg/m2), total energy intake (kcal/day), smoking status (current, former, never), physical activity (tertiles of MET-min/week), and NOVA classification system groups except group 4.  Bold indicates p-value<0.05. | | | | | |
